# Muscle Cramping During a 161-km Ultramarathon: Comparison of Characteristics of Those With and Without Cramping

**DOI:** 10.1186/s40798-015-0019-7

**Published:** 2015-05-21

**Authors:** Martin D Hoffman, Kristin J Stuempfle

**Affiliations:** 1 Department of Physical Medicine & Rehabilitation, Department of Veterans Affairs, Northern California Health Care System, and University of California Davis Medical Center, 10535 Hospital Way, Sacramento, CA USA; 2 Health Sciences Department, Gettysburg College, Gettysburg, PA USA

**Keywords:** Creatine kinase, Endurance exercise, Exercise, Muscle cramp, Muscle fatigue, Running, Sodium, Water-electrolyte imbalance

## Abstract

**Background:**

This work sought to identify characteristics differing between those with and without muscle cramping during a 161-km ultramarathon.

**Methods:**

In this observational study, race participants underwent body weight measurements before, during, and after the race; completed a post-race questionnaire about muscle cramping and “near” cramping (controllable, not reaching full-blown cramping), drinking strategies, and use of sodium supplementation during four race segments; and underwent a post-race blood draw for determination of serum sodium and blood creatine kinase (CK) concentrations.

**Results:**

The post-race questionnaire was completed by 280 (74.5 %) of the 376 starters. A post-race blood sample was provided by 181 (61.1 %) of the 296 finishers, and 157 (53.0 %) of finishers completed the post-race survey and also provided a post-race blood sample. Among those who completed the survey, the prevalence of cramping and near cramping was 14.3 and 26.8 %, respectively, with greatest involvement being in the calf (54 %), quadriceps (44 %), and hamstring (33 %) muscles. Those with cramping or near cramping were more likely to have a prior history of muscle cramping during an ultramarathon (*p* < 0.0001) and had higher blood CK concentrations (*p* = 0.001) than those without cramping. Weight change during the race, use of sodium supplements, intake rate of sodium in supplements, and post-race serum sodium concentration did not differ between those with and without cramping.

**Conclusions:**

Muscle cramping is most common in those with a prior history of cramping and greater muscle damage during an ultramarathon, suggesting an association with relative muscular demand. Impaired fluid and sodium balance did not appear to be an etiology of muscle cramping during an ultramarathon.

## Key points

- Muscle cramping and near cramping is common in a 161-km ultramarathon and largely involves the most active muscles (calves, quadriceps and hamstrings).
- Those with cramping or near cramping are more likely to have a prior history of muscle cramping during an ultramarathon and greater muscle damage during the event than those without cramping. Hydration status, intake rate of sodium in supplements and serum sodium concentration do not differ between those with and without cramping.
- The findings do not support an electrolyte depletion and/or dehydration basis for muscle cramping in ultramarathon running.

## Background

Exercise-associated cramping of skeletal muscles is common among participants in numerous sports and other physical activities [1–4]. For instance, one study showed that the prevalence of muscle cramping during or within 6 h after an Ironman triathlon was 23 % among study participants [5], and the prevalence was 41 % at a 56-km ultramarathon [6]. In another study, muscle cramping during or immediately after a 100-km ultramarathon was present in 23 % of study participants [7]. We have previously reported that muscle cramping was the main reason for dropping out of a 161-km ultramarathon by 5 % of those failing to finish, and muscle cramps were indicated to have adversely affected performance in 11 and 16 % of finishers and non-finishers, respectively [8]. Muscle cramping has also been shown to be a cause for 1–3 % of athletes to seek medical attention during ultra-endurance footraces [9, 10].

Despite the high prevalence and negative impact on performance from muscle cramping during endurance activities, controversy continues about the underlying pathophysiology [1–4, 11–13]. Two distinct theories have emerged for the cause of exercise-associated muscle cramps: (1) altered neuromuscular control from muscular fatigue resulting in increased excitatory and decreased inhibitory afferent inputs to motor neurons, and (2) hyperexcitability of motor neuron axon terminals induced by mechanical deformation and exposure to increased levels of excitatory extracellular constituents in the surrounding extracellular space [2, 12, 13]. The latter theory depends on a presumption that there is electrolyte depletion and/or dehydration. Since the prevention and treatment strategies are quite dissimilar between these potential underlying causes, further exploration into the pathophysiology of exercise-associated muscle cramping is important and carries practical implications.

Prior studies of muscle cramping associated with endurance activities up to ~18 h have shown a higher likelihood of cramping among those with a prior history of cramping [5, 6] and those racing at a relatively higher intensity [5, 6, 14]. Muscle cramping has not been related to serum sodium concentration [5, 15–17]. The purpose of this study was to learn about muscle cramping during an ultramarathon lasting up to 30 h, including the prevalence, muscle groups involved, distance at which cramping tends to occur, characteristics of those who have muscle cramping, and whether muscle cramping is related to weight change, sodium supplementation, post-race serum sodium, or extent of muscle injury as defined by serum creatine kinase (CK) concentration. It was anticipated that this new information would offer additional insight into the underlying pathophysiology of exercise-associated muscle cramping in this environment. Based on prior findings from endurance events [2–6, 8, 15–18], we hypothesized that muscle cramping would be common and more likely among those with a prior history of muscle cramping and is unrelated to hydration status, intake rate of sodium in supplements, or post-race serum sodium concentration.

## Methods

The study was performed at the 2014 Western States Endurance Run, a 161.3-km ultramarathon through the Sierra Nevada Mountains of Northern California with 5500 m of cumulative climb and 7000 m of cumulative descent. Additional race details have been provided elsewhere [8, 19–23]. Nearby ambient temperatures during the competition ranged from a low of 0 °C just after the start to a high of 31.7 °C in the afternoon, which was close to the historical median high temperature for this event, but our on-course measurements (Vantage Vue Wireless Weather Station, Davis Instruments, Vernon Hills, IL) recorded a maximum air temperature of 39 °C at which time the relative humidity was 13 %. The research was approved by the institutional review boards of the Veterans Affairs Northern California Health Care System and Gettysburg College with electronic consent obtained from those participating in the questionnaire.

Body weight measurements were performed on all race participants within 1.5 h before the start of the race; when reaching the 47.8, 89.6, and 125.5 km aid stations during the race; and again immediately after finishing the race. All weight measurements were made with calibrated scales (Health O Meter, model 349KLX, Boca Raton, FL) that were on firm, level surfaces. The runners were clothed in running wear and shoes, but jackets and other items such as waist packs and hydration vests were removed, and nothing was permitted in the runner’s hands. Prior to the event, the scales were examined for consistency, and though the maximum variation between scales was less than 0.5 % across the weight range of our subjects, correction equations were developed to standardize all weight measurements to a single scale.

Blood samples were drawn from willing runners within a few minutes after finishing the race. Runners were seated while blood was drawn into heparinized tubes via an antecubital vein. Samples were stored in a cooler until analyzed by a clinical laboratory for serum sodium, blood urea nitrogen (BUN), creatinine, and CK concentrations (Seimens Aktiengesellschaft, Dimension EXL, Munich, Germany).

Race participants were alerted before the race that they would be asked to complete a post-race web-based questionnaire. The invitation to the questionnaire was sent electronically to all race starters during the event. Those who had not completed the survey were sent reminder emails 7 and 12 days later, and the survey was closed 15 days after the race. The questionnaire requested information on running background and training during the 3 months prior to the race. It requested the number and brand of sodium supplements used (if any) during each of four segments of the race defined by the sites where body weights were measured. A list of the most commonly used commercially available products was provided, and the runner also had the opportunity to specify other forms of sodium supplementation. From the known sodium content of each brand of sodium supplement and official split times, the rate of sodium intake in supplements was calculated.

The questionnaire also asked whether or not the runner had experienced muscle cramping during prior ultramarathons and if they had had muscle cramping during this event, with answer options of yes, no, and “almost, but was able to control any full-blown cramping” (subsequently referred to as “near cramping”). The answer option of near cramping was included because we are aware that runners sometimes sense impending muscle cramping that can be aborted with various interventions, such as altering gait pattern. Those with cramping or near cramping were asked which muscle groups were involved and in which of the four course segments they experienced the cramping.

Between-group comparisons of categorical data were made with Fisher’s exact test or chi-square test. Continuous data underwent normality testing with the D’Agostino-Pearson test. Group comparisons of continuous data were made with one-way analysis of variance and Tukey post-test, or the Kruskal-Wallis test and Dunn’s multiple comparison test depending on whether or not the data passed normality testing. When two groups were compared, the unpaired *t* test or Mann Whitney test was used depending on findings from normality testing. Spearman correlation analysis was used for the single correlation performed since the data were not normally distributed. Statistical significance was set at *p* < 0.05.

## Results

The race had 376 starters and 296 (78.7 %) finishers. The post-race survey was completed by 280 (74.5 %) of the starters. Survey completion rate was similar (*p* = 0.46) between men and women, but was greater (*p* = 0.0005) among finishers (78.7 %) than non-finishers (58.8 %). Of those completing the survey, 55.7 % did so within 7 days of the end of the race and 88.9 % did so within 10 days. A post-race blood sample was provided by 181 (61.1 %) finishers. There were 157 (53.0 %) finishers who completed the post-race survey and also provided a post-race blood sample.

**Fig 1:**
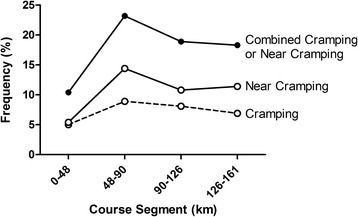
Frequencies of muscle cramping, near cramping, and either cramping or near cramping (combined cramping or near cramping) during the four course segments. Frequencies are shown relative to the number of race participants who ran the specified course segment. Within groups, there were no differences in frequencies across course segments

Muscle cramping at some time during the race was reported by 40 (14.3 %) runners completing the survey. An additional 75 (26.8 %) indicated they had experienced near cramping. The frequencies of muscle cramping, near cramping, and either cramping or near cramping during the four course segments are shown in Fig. 1. The frequency of cramping (5.0–8.9 %) and near cramping (5.4–14.4 %) did not vary across segments (*p* = 0.99 and *p* = 0.64, respectively). The frequency of combined cramping or near cramping (10.4–23.2 %) was also not different across segments (*p* = 0.35).

The frequency distribution of muscle cramping in different body locations or muscle groups is shown in Table 1. The calf, quadriceps, and hamstrings were the most common muscle groups involved. Involvement of the quadriceps and forearm was less common (*p* < 0.05) for those with near cramping than for those with cramping.

**Table 1:** Frequency of muscle cramping in different body locations or muscle groups among those with cramping (n = 40), those with near cramping (n = 75), and those with either cramping or near cramping (n = 115)

Body location or muscle group	Combined cramping or near cramping (%)	Cramping (%)	Near cramping (%)	*p* value
Calf	53.9	57.5	52.0	0.69
Quadriceps	43.5	57.5	36.0	0.031
Hamstrings	33.0	45.0	26.7	0.061
Hip flexors	15.7	17.5	14.7	0.79
Trunk	6.1	10.0	4.0	0.23
Hip adductors	4.3	2.5	5.3	0.66
Ankle dorsiflexors	3.5	7.5	1.3	0.12
Forearm	2.6	7.5	0.0	0.040
Feet	2.6	5.0	1.3	0.28
Upper arm	1.7	2.5	1.3	1.00
Hands	0.9	2.5	0.0	0.35

Values are expressed as percentages of those reporting they had experienced muscle cramping or near cramping. Since some runners had multiple areas with muscle cramping, the summation of percentages is greater than 100 %. The statistical comparison is between the cramping and near cramping groups for each body location or muscle group

Characteristics of runners with cramping, near cramping, and no cramping are compared in Table 2. These three groups did not differ by age, sex, ultramarathon running experience, number of prior 161-km ultramarathon running finishes or drops, running training distance, finish status or time, use of sodium supplementation, rate of sodium intake in supplements, minimum weight during the race, and post-race serum sodium and blood creatinine. A prior history of muscle cramping during an ultramarathon was more common among those with cramping (*p* = 0.0013) and near cramping (*p* < 0.0001) than those with no cramping. In fact, those with a prior history of muscle cramping during an ultramarathon were 2.5 times more likely to have had cramping or near cramping during the present event than those without a prior history of muscle cramping (94 of 180 or 52.2 % compared with 21 of 100 or 21.0 %). Post-race BUN and blood CK concentrations were higher for the cramping group than the group without cramping (*p* < 0.05 and *p* < 0.001, respectively), as well as for the combined cramping or near cramping group compared with the group without cramping (*p* = 0.030 and 0.0013, respectively). Finish time was also slower (*p* = 0.048) for the combined cramping or near cramping group compared with the no cramping group.

**Table 2:** **Comparison of characteristics among runners with cramping, near cramping, and no cramping, as well either cramping or near cramping**

Variable	Combined cramping or near cramping	Cramping	Near cramping	No cramping	*p* value^a^
Age (years)	42 ± 9	43 ± 10	42 ± 9	42 ± 9	0.84
Sex (*n*, % men)	97, 84.3	32, 80.0	65, 86.7	126, 76.4	0.18
Ultramarathon running experience (years)	5 (3–8)	4 (3–8)	5 (3–8)	5 (3–9)	0.45
Prior 161-km ultramarathon finishes (*n*)	2 (1–4)	1 (1–3)	2 (1–4)	2 (0–5)	0.46
Prior 161-km ultramarathon drops (*n*)	0 (0–1)	0 (0–1)	0 (0–1)	0 (0–2)	0.94
Average running distance (km/week)^b^	97 (80–113)	89 (80–120)	97 (75–113)	97 (80–121)	0.70
Highest running distance in 1 week (km)^b^	145 (125–169)	142 (124–167)	145 (126–169)	140 (118–166)	0.61
Longest single run (km)^b^	80 (68–100)	80 (56–100)	80 (72–100)	80 (65–100)	0.46
Prior history of cramping (*n*, %)	94, 81.7^†^	32, 80.0*	62, 82.7*	86, 52.1	<0.0001
Finish status (*n*, % finished)	97, 84.3	31, 77.5	66, 88.0	136, 82.4	0.33
Finish time (h)	26.52 (23.37–28.61)^†^	26.60 (22.02–28.83)	26.41 (23.56–28.48)	25.06 (22.16–27.98)	0.14
Sodium supplementation (*n*, % using)	109, 94.8	38, 95.0	71, 94.7	154, 93.3	0.88
Sodium supplement intake (mg/h)	145 (59–295)	128 (35–261)	147 (66–328)	132 (62–235)	0.46
Minimum weight (% of start weight)	−3.0 (−4.3 to −2.0)	−3.3 (−4.4 to −2.5)	−2.9 (−4.0 to −1.9)	−3.1 (−4.1 to −2.1)	0.23
Post-race serum sodium (mmol/L)	139 ± 4	138 ± 5	140 ± 4	140 ± 3	0.055
Post-race blood CK (IU/L)	19,458 (10,098–46,887)^†^	35,265 (14,755–53,567)*	15,149 (9120–38,920)	11,862 (5951–24,615)	0.0010
Post-race BUN (mg/dL)	29 (22–36)^†^	35 (26–39)*	29 (20–34)	25 (20–33)	0.023
Post-race blood creatinine (mg/dL)	1.30 (1.12–1.53)	1.32 (1.15–1.60)	1.26 (1.04–1.48)	1.18 (1.03–1.41)	0.19

Values are reported as mean ± SD when data were normally distributed for the cramping, near cramping, and no cramping groups; median and interquartile range if not normally distributed for these groups; or group count (*n*) and percentage within the study group providing data for the variable

*Indicates statistical difference on post-test compared with the no cramping group

^†^Indicates statistical difference in the two-group comparison with the no cramping group

^a^ *p* value for the comparison of cramping, near cramping, and no cramping groups

^b^During the 3 months prior to the event

Post-race serum sodium was further compared among those with cramping (mean ± SD value 138 ± 5 mmol/L), near cramping (140 ± 4 mmol/L), and no cramping (140 ± 3 mmol/L) during the last segment of the race and was found to not be different among groups (*p* = 0.51). Additionally, the proportion of runners with a serum sodium concentration at the finish below 135 mmol/L (reference range for normal is 135–145 mmol/L) was similar (*p* = 0.43) for those with cramping or near cramping (11.5 %) and those without cramping (7.0 %) during the final segment of the race, and the serum sodium concentrations for these two groups (range 125–133 and 131–134 mmol/L, respectively) were also similar (*p* = 0.16).

**Fig 2:**
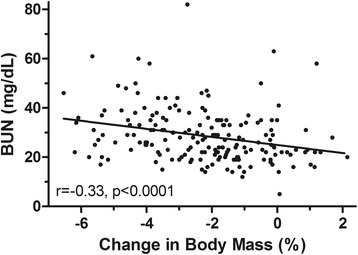
Relationship of post-race BUN concentration with percent change in body weight from the start to finish

The relationship of post-race BUN with change in body weight is shown in Fig. 2. A significant inverse relationship (*r* = −0.33, *p* < 0.0001) was identified.

**Fig 3:**
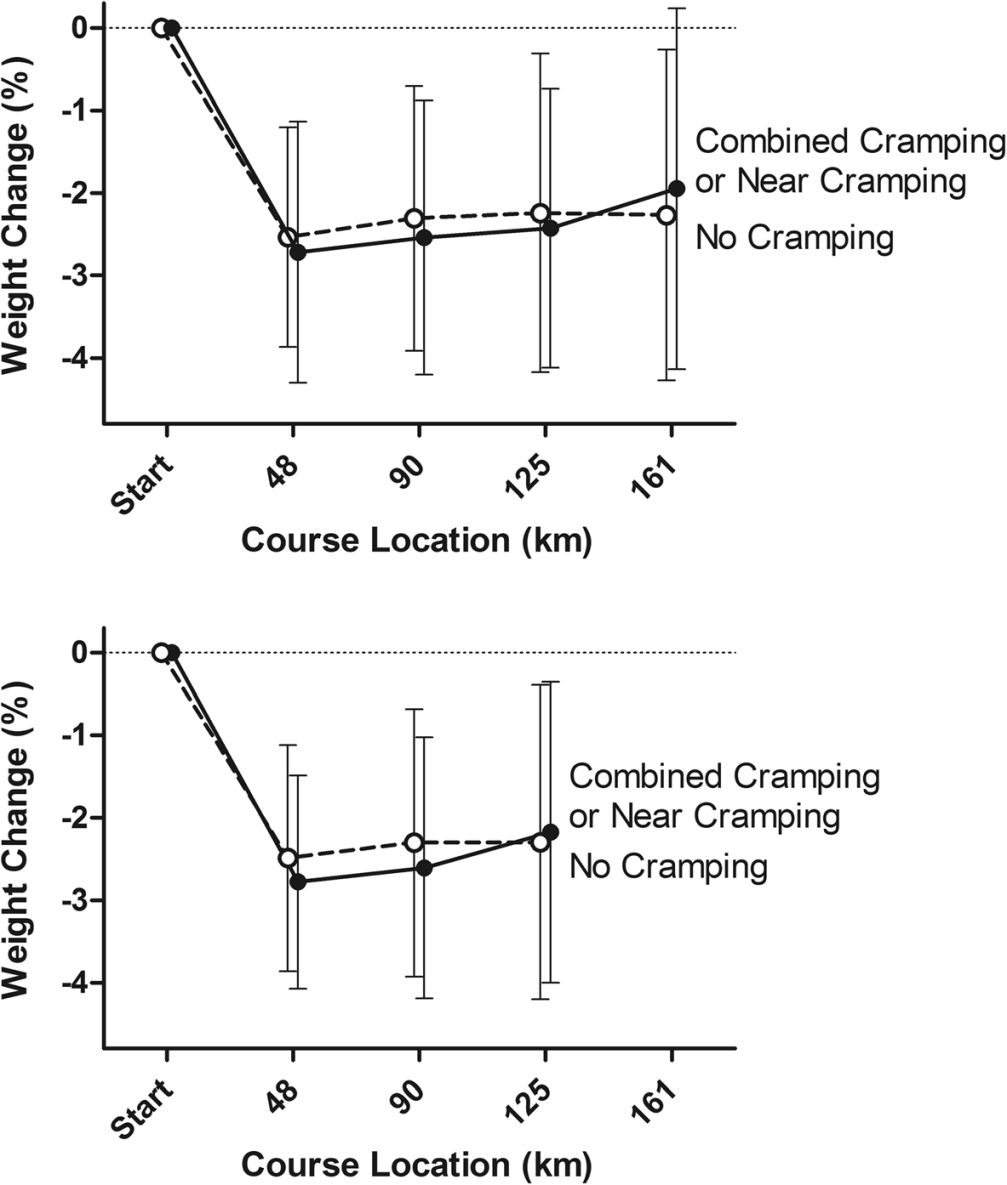
Mean weight change at course locations where weights were obtained for those with either cramping or near cramping (*closed circles* and *solid lines*) and those without cramping (*open circles* and *dashed lines*) during the race segment ending where weight was measured (*upper graph*) and during the subsequent course segment (*lower graph*). Data include finishers and non-finishers. *Error brackets* represent 1 SD. Data points are offset slightly along the horizontal axis for clarity of the error brackets

Comparison of weight change across the course between those with cramping or near cramping and those without cramping is shown in Fig. 3. Weight change did not differ between groups whether comparing the weight change at the end of the course segment in which the symptoms were considered or the weight change at the end of the segment prior to that for which the symptoms were considered.

**Fig 4:**
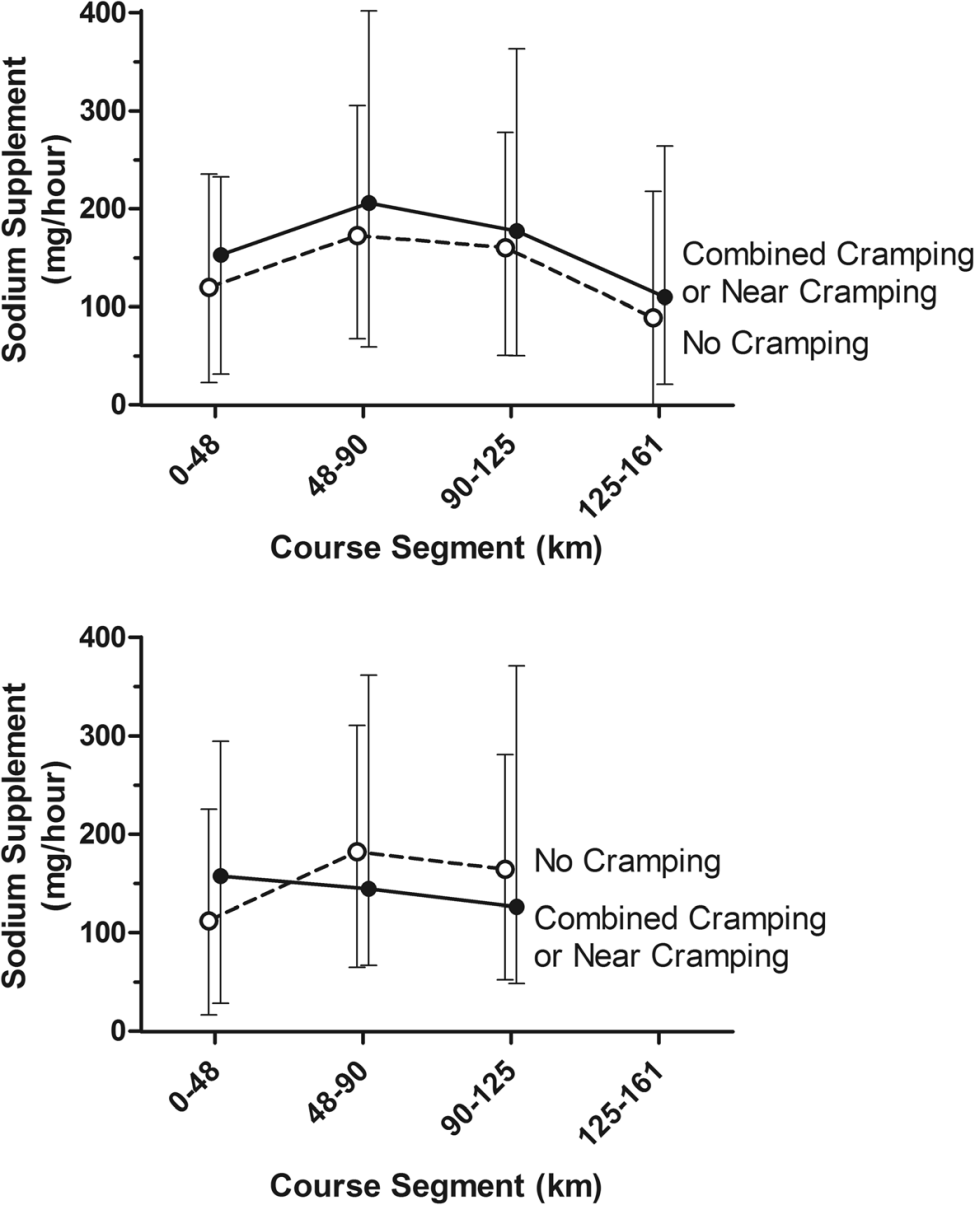
Median rate of intake of sodium in supplements during each course segment for those with either cramping or near cramping (*closed circles* and *solid lines*) and those without cramping (*open circles* and *dashed lines*) during the same segment (*upper graph*) and during the subsequent segment (*lower graph*). Data include finishers and non-finishers. *Error brackets* represent interquartile range. Data points are offset slightly along the horizontal axis for clarity of the error brackets

Comparison of rate of sodium intake in supplements during each course segment between those with cramping or near cramping and those without cramping is shown in Fig. 4. The rate of sodium intake in supplements did not differ between groups whether comparing intake during the course segment where cramping or near cramping occurred or the intake during the prior segment. Two runners with cramping and two with near cramping reported taking sodium in supplements at an average rate of 772–872 mg/h. Both runners also reported they were consuming electrolyte-containing energy drinks. Also, 12 runners (11 finishers) without cramping were taking no sodium in supplements throughout the race, and 1 (a finisher) indicated he also used no electrolyte-containing energy drinks for hydration purposes.

## Discussion

The key findings of this study are that (1) cramping and near cramping were common in this 161-km ultramarathon; (2) the most commonly involved muscles were the calf, quadriceps, and hamstrings; (3) those with cramping or near cramping were more likely to have a prior history of muscle cramping during an ultramarathon and greater muscle damage during the race than those without cramping; and (4) hydration status, intake rate of sodium in supplements, and serum sodium concentration did not differ between those with cramping and those without cramping.

Prior work has demonstrated that muscle cramping is common in 161-km ultramarathons [8]. Prevalence rates of muscle cramping during or within 6 h after an Ironman triathlon were reported at 23 % [5], and the prevalence rate was 41 % at a 56-km ultramarathon [6]. While the present study did not include a post-race assessment for muscle cramping prevalence, and considered both cramping and near cramping, the 14 % prevalence of cramping and 41.1 % prevalence with inclusion of those with near cramping offer further support for this being a common phenomenon in ultramarathon running.

Muscle cramping was most commonly experienced in the large muscle groups primarily involved in running (i.e., calf, quadriceps, hamstrings) and subject to overload and fatigue during an ultramarathon. The lesser involved muscle groups and body locations could have also been subject to fatigue. This would even be true for muscles in the upper extremity, especially considering that many of the runners in these events carry fluid bottles in their hands while running. Nevertheless, since most of the cramping involved those muscle groups primarily subject to fatigue, a mechanism for cramping that involves muscle overload and fatigue seems to be supported.

Unfortunately, a formal assessment of muscular fatigue was not made in this study, so the extent of muscular fatigue across the course is not known. Although progressive muscular fatigue might be expected during a continuous event, that does not appear to be the case as muscular fatigue has been found to stabilize by around 12 h during a 24-h treadmill run [24]. Indeed, runners may accommodate by slowing as has been previously demonstrated to begin around 70 km in the present event [22, 25] and after approximately 6 h during a 24-h treadmill run [24, 26]. Variation in course profile, ambient temperature conditions, visibility impairment from darkness, and available energy sources contribute to these changes in speed during the Western States Endurance Run, making it difficult to know the extent of muscular fatigue throughout the event. Such factors may contribute to our finding that muscle cramping did not vary statistically across the course segments, although inspection of the data suggests that cramping and near cramping tended to be most common after the first course segment.

Runners developing muscle cramping during the ultramarathon were distinguished from those without cramping by more commonly having a prior history of muscle cramping during an ultramarathon and having higher post-race blood CK and BUN concentrations. A prior history of muscle cramping has previously been found to be a characteristic of those developing cramping during endurance events [5, 6], so this finding is not surprising. However, as far as we are aware, higher blood CK and BUN concentrations among those with cramping are new findings. While numerous factors account for variability in post-race blood CK concentrations, our interpretation of the finding that those with cramping had a higher blood CK relates to an association of muscle injury with relative exercise demand. In other words, we presume that the higher CK concentrations indicate that the group with cramping was placing greater demands on their muscles during the race relative to their current state of training than those without cramping. This provides additional support to the prior observation that those developing muscle cramping are racing at a relatively higher intensity than those without cramping [5, 6, 14] and that cramping in this environment may be due to muscle overload and fatigue rather than a fluid and/or electrolyte imbalance.

Interpretation of the present finding of a higher BUN concentration among those with cramping compared with those without cramping requires some exploration. In general, BUN concentration is related to hydration status. Similar to the present work, we have previously demonstrated a relationship between BUN and change in body weight such that BUN is higher among those with greater body weight loss during the event [27]. Yet for that study and the present study, weight change only explained 11–13 % of the variability of BUN concentration. It is likely because of this relatively weak relationship that the present work showed no evidence that cramping was related to percent weight change. In other words, while the post-race BUN concentrations were statistically higher among those with cramping than those without cramping, closer inspection of hydration status by weight change showed no evidence that those with cramping were more likely to have an issue with fluid balance. This finding that muscle cramping is not related to hydration level supports prior findings from observational studies of runners and triathletes [5, 15–17] as well as controlled laboratory studies [28, 29].

Runners developing muscle cramping during the ultramarathon did not have a statistically different post-race serum sodium compared with those without cramping, but the statistical effect was close to significant (*p* = 0.055). To further investigate whether a low serum sodium (below the reference range for normal of 135–145 mmol/L) might be linked to a higher incidence of cramping, we performed additional analyses. The serum sodium concentration among those with cramping, near cramping, and no cramping during the final segment of the race was compared and found to not be different. We also compared the proportion of runners with serum sodium concentrations below normal among those with cramping or near cramping and those without cramping during the final segment of the race and found no difference between groups. These analyses were performed under the presumption that the serum sodium concentration at the finish would relate best to any symptoms during the final segment of the race. Given the findings from these analyses, we conclude that cramping is not related to serum sodium concentration in this environment, a conclusion that supports prior studies of runners and triathletes [5, 15–17].

The use of sodium supplementation is common during 161-km ultramarathons. In fact, our prior work at this same event has demonstrated that 90–96 % of runners use sodium supplements [30, 31], which is comparable to the overall use by 94 % in the present work. Despite such high use of sodium supplements, we have found that they have little or no effect on hydration status [30] or the prevention of hyponatremia [31, 32]. Furthermore, total sodium intake has no relationship with the common symptoms of nausea and vomiting [33]. The present work further demonstrates that the use of sodium supplements and the rate of intake of sodium in supplements are not related to muscle cramping. Interestingly, it has been shown that pickle juice can inhibit electrically induced muscle cramps in mildly dehydrated humans; however, the effect was evident before absorption could have occurred [34]. It was speculated that some component (not necessarily the electrolyte content) of the pickle juice might trigger a reflex from the oropharyngeal region that inhibits alpha motor neurons [34].

The present findings offer some insight into the underlying pathophysiology of exercise-associated muscle cramping. Key findings in this regard were that (1) the cramping involved muscle groups under high demand during the activity; (2) cramping appeared most prevalent, though not statistically different, after the early portion of the race; (3) those with cramping had higher blood CK concentrations than those without cramping; and (4) cramping was not related to serum sodium concentration or hydration status. Considering the two main theories for the pathophysiology of exercise-associated muscle cramping, these findings seem most consistent with muscle fatigue as an underlying mechanism for muscle cramping in this environment rather than an electrolyte or fluid balance issue.

The theory of exercise-associated cramping being due to hyperexcitability of motor neuron axon terminals relies on the premise that there is a loss in plasma volume during exercise from sweating that causes a shift of water from the interstitial compartment to the intravascular space [12]. Increased local concentrations of excitatory extracellular constituents such as acetylcholine, electrolytes, and exercise-related metabolites are thought to then provide an environment for hyperexcitability of neuromuscular junctions. While plasma volume was not measured in the present study, the findings of similar serum sodium concentrations and weight changes between those with and without cramping suggest that there was no difference in the level of plasma volume contraction or fluid shift from the interstitial compartment between groups.

The study is limited in some regards, largely due to the restraints of performing research at a competitive event. Such work generally requires an observational design, which makes it challenging to specifically examine underlying pathophysiology. Furthermore, since the current beliefs among most participants in the present event is that sodium supplementation is important, we had to accept that most subjects would be using sodium supplements. This means that the number of subjects with muscle cramping who did not use sodium supplements was small. We were also limited by an inability to quantify total sodium intake which requires a full dietary analysis and is not feasible with a large sample size. Another limitation is that the study depended on subject willingness to provide a post-race blood sample and to complete the post-race questionnaire. The latter depended on subject recall, although runners were alerted in advance that they would be asked to provide information about hydration strategies. Furthermore, significant memory distortion was likely limited since most study participants completed the survey within a few days of the race and since most runners avoid adopting new hydration approaches for an event of this nature. Because the study was performed at a competitive event, the blood work was only performed at the finish, although it would have been optimal to know the intermediate values in order to more closely examine relationships with cramping during each race segment. Finally, a formal measurement of muscular fatigue during each race segment would have been valuable, but was not feasible on a large scale in a competitive event.

## Conclusions

From this work, we conclude that muscle cramping and near cramping are common in a 161-km ultramarathon, largely involve the most active muscles, and tend to occur after the early segment of the race. Compared with those not having cramping, those with cramping or near cramping are more likely to have a prior history of muscle cramping during an ultramarathon, and evidence suggests that they are placing greater demands on their muscles during the race relative to their current state of training. Hydration status, intake rate of sodium in supplements, and serum sodium concentration do not differ between those with and without cramping. Of the two main theories for the pathophysiology of exercise-associated muscle cramping, the present findings support a muscle fatigue basis for muscle cramping during ultra-endurance exercise over the theory based on hyperexcitability of motor neuron axon terminals due to an electrolyte or fluid imbalance.

## Abbreviations

BUN: Blood urea nitrogen
CK: Creatine kinase

## References

[1] Eichner ER (2007). The role of sodium in ‘heat cramping’. Sports Med.

[2] Schwellnus MP (2009). Cause of exercise associated muscle cramps (EAMC)—altered neuromuscular control, dehydration or electrolyte depletion?. Br J Sports Med.

[3] Schwellnus MP (2007). Muscle cramping in the marathon: aetiology and risk factors. Sports Med.

[4] Schwellnus MP, Drew N, Collins M (2008). Muscle cramping in athletes—risk factors, clinical assessment, and management. Clin Sports Med.

[5] Schwellnus MP, Drew N, Collins M (2011). Increased running speed and previous cramps rather than dehydration or serum sodium changes predict exercise-associated muscle cramping: a prospective cohort study in 210 Ironman triathletes. Br J Sports Med.

[6] Schwellnus MP, Allie S, Derman W, Collins M (2011). Increased running speed and pre-race muscle damage as risk factors for exercise-associated muscle cramps in a 56 km ultra-marathon: a prospective cohort study. Br J Sports Med.

[7] Kao WF, Hou SK, Chiu YH, Chou SL, Kuo FC, Wang SH (2015). Effects of 100-km ultramarathon on acute kidney injury. Clin J Sport Med.

[8] Hoffman MD, Fogard K (2011). Factors related to successful completion of a 161-km ultramarathon. Int J Sports Physiol Perform.

[9] McGowan V, Hoffman MD. Characterization of medical care at the 161-km Western States Endurance Run. Wilderness Environ Med 2015;26:29–35 10.1016/j.wem.2014.06.015 25281587

[10] Scheer BV, Murray A (2011). Al Andalus Ultra Trail: an observation of medical interventions during a 219-km, 5-day ultramarathon stage race. Clin J Sport Med.

[11] Eichner ER (2014). The salt paradox for athletes. Curr Sports Med Rep.

[12] Bergeron MF (2008). Muscle cramps during exercise—is it fatigue or electrolyte deficit?. Curr Sports Med Rep.

[13] Minetto MA, Holobar A, Botter A, Farina D (2013). Origin and development of muscle cramps. Exerc Sport Sci Rev.

[14] Shang G, Collins M, Schwellnus MP (2011). Factors associated with a self-reported history of exercise-associated muscle cramps in Ironman triathletes: a case-control study. Clin J Sport Med.

[15] Maughan RJ (1986). Exercise-induced muscle cramp: a prospective biochemical study in marathon runners. J Sports Sci.

[16] Schwellnus MP, Nicol J, Laubscher R, Noakes TD (2004). Serum electrolyte concentrations and hydration status are not associated with exercise associated muscle cramping (EAMC) in distance runners. Br J Sports Med.

[17] Sulzer NU, Schwellnus MP, Noakes TD (2005). Serum electrolytes in Ironman triathletes with exercise-associated muscle cramping. Med Sci Sports Exerc.

[18] Schwellnus MP, Derman EW, Noakes TD (1997). Aetiology of skeletal muscle ‘cramps’ during exercise: a novel hypothesis. J Sports Sci.

[19] Hoffman MD, Ingwerson JL, Rogers IR, Stuempfle KJ, Hew-Butler T (2012). Increasing creatine phosphokinase concentration at the 161-km Western States Endurance Run. Wilderness Environ Med.

[20] Hoffman MD, Stuempfle KJ, Rogers IR, Weschler LB, Hew-Butler T (2011). Hyponatremia in the 2009 161-km Western States Endurance Run. Int J Sports Physiol Perform.

[21] Hoffman MD, Wegelin JA (2009). The Western States 100-Mile Endurance Run: participation and performance trends. Med Sci Sports Exerc.

[22] Parise C, Hoffman MD (2011). Influence of temperature and performance level on pacing a 161-km trail ultramarathon. Int J Sports Physiol Perform.

[23] Rogers IR, Hook G, Stuempfle KJ, Hoffman MD, Hew-Butler T (2011). An intervention study of oral versus intravenous hypertonic saline administration in ultramarathon runners with exercise-associated hyponatremia: a preliminary randomized trial. Clin J Sports Med.

[24] Martin V, Kerhervé H, Messonnier LA, Banfi JC, Geyssant A, Bonnefoy R (2010). Central and peripheral contributions to neuromuscular fatigue induced by a 24-h treadmill run. J Appl Physiol (1985).

[25] Hoffman MD (2014). Pacing by winners of a 161-km mountain ultramarathon. Int J Sports Physiol Perform.

[26] Gimenez P, Kerhervé H, Messonnier LA, Féasson L, Millet GY (2013). Changes in the energy cost of running during a 24-h treadmill exercise. Med Sci Sports Exerc.

[27] Hoffman MD, Stuempfle KJ, Fogard K, Hew-Butler T, Winger J, Weiss RH (2013). Urine dipstick analysis for identification of runners susceptible to acute kidney injury following an ultramarathon. J Sports Sci.

[28] Braulick KW, Miller KC, Albrecht JM, Tucker JM, Deal JE (2013). Significant and serious dehydration does not affect skeletal muscle cramp threshold frequency. Br J Sports Med.

[29] Miller KC, Mack GW, Knight KL, Hopkins JT, Draper DO, Fields PJ (2010). Three percent hypohydration does not affect threshold frequency of electrically induced cramps. Med Sci Sports Exerc.

[30] Hoffman MD, Stuempfle KJ (2014). Hydration strategies, weight change and performance in a 161 km ultramarathon. Res Sports Med.

[31] Winger JM, Hoffman MD, Hew-Butler TD, Stuempfle KJ, Dugas JP, Fogard K (2013). Physiology and hydration beliefs affect race behavior but not post-race sodium in 161-km ultramarathon finishers. Int J Sports Physiol Perform.

[32] Hoffman MD, Stuempfle KJ. Sodium supplementation and exercise-associated hyponatremia during prolonged exercise. Med Sci Sports Exerc 2014. [Epub ahead of print] 10.1249/MSS.0000000000000599 25551404

[33] Stuempfle KJ, Hoffman MD, Weschler LB, Rogers IR, Hew-Butler T (2011). Race diet of finishers and non-finishers in a 100 mile (161 km) mountain footrace. J Am Coll Nutr.

[34] Miller KC, Mack GW, Knight KL, Hopkins JT, Draper DO, Fields PJ (2010). Reflex inhibition of electrically induced muscle cramps in hypohydrated humans. Med Sci Sports Exerc.

